# Mothers’ interoceptive sensibility mediates affective interaction between mother and infant

**DOI:** 10.1038/s41598-022-09988-y

**Published:** 2022-04-15

**Authors:** Ayami Suga, Yosuke Naruto, Venie Viktoria Rondang Maulina, Maki Uraguchi, Yuka Ozaki, Hideki Ohira

**Affiliations:** 1 Unicharm Corporation, 1531-7, Wadahama Toyohama-cho, Kanonji-shi, Kagawa, 769-1602 Japan; 2 Department of Psychology, Nagoya University, Nagoya, 464-8601 Japan; 3 Department of Psychology, Atma Jaya Catholic University of Indonesia, Jakarta, Indonesia; 4 Department of Social Psychology, Toyo University, Tokyo, 112-8606 Japan

**Keywords:** Psychology, Human behaviour

## Abstract

Interoceptive sensibility, which denotes the self-perceived sensitivity to inner-body sensations, has been associated with the emotional experiences and inferences of others’ emotional states. Focusing on the role of interoceptive sensibility in the emotional states and psychological well-being of mothers during caregiving, this study explores how physiological arousal and interoceptive sensibility mediate the association between mother–infant interaction and maternal well-being using an experience sampling method. Infant-directed-singing (IDS) with social touch was used to facilitate mother–infant interaction. Pairs of 2–8-month-old infants and their mothers participated. Mothers in an IDS group (*N* = 25) and a no-IDS group (*N* = 26) recorded their and the infant’s daily feelings and physiological states using a smartphone application for one month. All participants, including the control group (*N* = 78) who neither performed IDS nor used the application, answered the Multidimensional Assessment of Interoceptive Awareness questionnaire before and after the recording period. Results indicated that IDS improved mother–infant interactions and positive feelings, enhancing maternal physiological arousal. Increased interoceptive sensibility enhanced infants’ positive feelings in the IDS group, whereas in the no-IDS group, it weakened mother’s positive feelings, suggesting that maternal interoceptive sensibility mediated the effects of IDS on mother and infant well-being.

## Introduction

Behavioral interactions between infants and caregivers, such as touching, mutual gazing, and vocalizations, are important factors that influence infants’ development of language, social, and cognitive abilities^1^. Socio-emotional competencies develop through the physiological and social interactions between caregivers and infants in daily parenting^2^. The emotional well-being of the mother strongly influences how the mother–infant interaction facilitates adaptive infant development. Maternal mental health problems, such as postpartum depression and anxiety, can interfere with the quality of mother–infant interactions by decreasing maternal touch^3–6^.

Infant directed-singing (IDS) and social touch have been suggested as effective ways to enhance mother–infant interactions as well as to improve mothers’ well-being. Engaging in IDS correlates with increased maternal positive emotions, reduced physiological stress, and increased mother–infant proximity^7,8^. Social touch between mothers and infants is also associated with reduced physiological and emotional stress and postpartum depression in mothers^9,10^. Additionally, social touch is linked with the improved physical and cognitive development of infants^11,12^, promoting the development of physical self-awareness^13–15^. While the associations between IDS, social touch, and improvement in mother–infant interaction and mothers’ well-being have been primarily observed through correlational studies, more robust evidence that directly intervenes in real-life parenting is required.

Mothers’ physiological arousal and sensitivity to internal states (i.e., interoception) may well influence (i.e., mediate) the ability of IDS and social touch to improve mother–infant interactions and maternal well-being. Consistent with a long tradition in psychological theories of emotions^16,17^, physiological arousal measured by indices such as heart rate (HR) and skin conductance level during everyday life has been linked to the experiential valence (pleasant–unpleasant) of encountered events^18,19^. Interoception refers to sensations of physiological inner-body states and is considered as the basis of subjective emotional states, emotional experiences, and the processing of emotional stimuli^20–23^. Further, interoception is associated with sensitivity to others’ emotions and social emotions (empathy)^24–26^. Interoceptive sensibility is one of three subdivisions of interoception proposed by Garfinkel et al.^27^ and refers to the self-reported tendency to be interoceptive cognizant. Additionally, interoceptive sensibility has been reported to be highly associated with emotional identification and regulation^28–30^. The interoceptive sensibility of mothers and its association with physiological arousal might facilitate inferences about their infants’ physiological and emotional state as well as the infants’ ability to form accurate perceptions of bodily sensations^14^. This argument was supported by the findings that the parents’ interoceptive sensibility is associated with the child’s somatic symptoms through their sensitive caregiving behavior^31^ and that mothers’ interoceptive knowledge is associated with children’s emotion regulation and social skills^32^. As interoceptive ability determines the direction (positive/negative) and intensity of the influences of physiological arousal on affective states^33,34^, the interplay between interoception and arousal in caregivers might affect their attitudes toward infants and their experiences of affective states during caregiving.

Based on the argument above, this study examined whether IDS and social touch improved mother–infant interaction and reduced maternal distress, specifically perceived stress, and postnatal mood states in daily life by using the experience sampling method (ESM)^35^. If the combination of IDS and social touch can be confirmed to be effective in routine caregiving, it will help in developing intervention techniques that can be used on a daily basis. ESM is a method that instantly captures emotional and behavioral experiences and physiological responses in daily life, providing ecologically valid data^35,36^. Using this method, it was possible to grasp mother–infant interactions and physiological states.

For this study, a smartphone application was produced to track moment-to-moment experiences of the feelings (i.e., calmness, pleasantness, relaxation, and vitality) of healthy mothers and infants without a diagnosis of mental illness or cardiovascular diseases as well as the mothers’ HRs during daily caregiving. This study investigated whether interoceptive sensibility mediated the effect of mother–infant interactions on maternal well-being (affective state) in mothers who were assigned to an IDS or no-IDS form of mother–infant interaction (during diaper changing). Diaper changing is a routine and frequent caregiving task as well as an important opportunity for mother–infant interaction^37^. Therefore, we created a song to encourage maternal social touch during diaper changing and used it in this experiment. To enhance interoceptive sensibility, participants used a smartphone application that registered the HR at designated times (ESM). A control group of mothers who performed neither IDS nor ESM was assessed twice to determine the effects of repeated assessment per se on spontaneous changes in maternal affective states. It was hypothesized that mothers using IDS would experience increased positive interactions with increased interoceptive sensibility and report increased positive feelings compared with the no-IDS group. Notably, this study’s data collection was conducted before the COVID-19 pandemic.

## Results

### Behaviors and psychological variables and interoceptive sensibility

**Figure 1:**
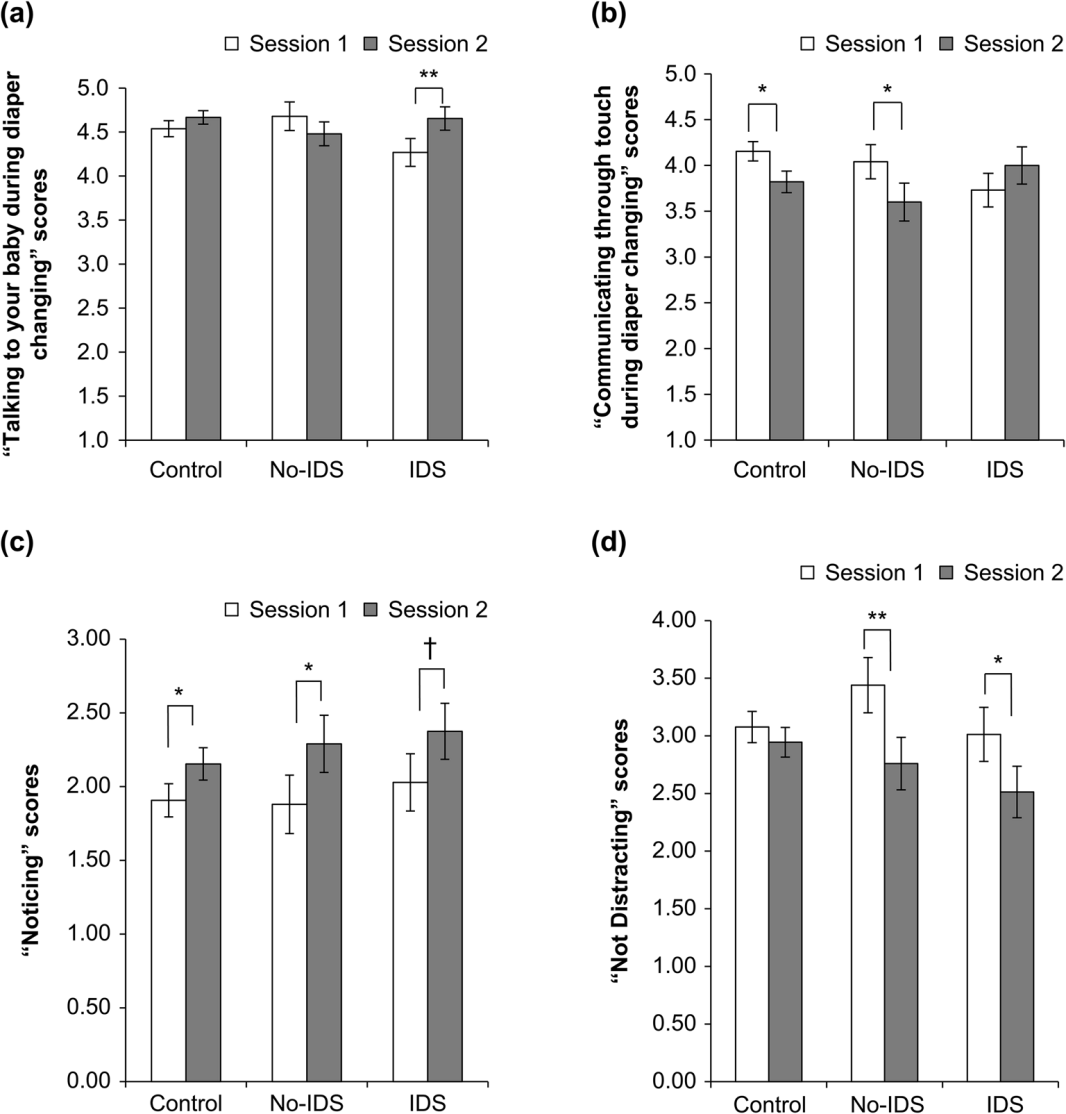
Behaviors and interoceptive sensibility scores from Session 1 to Session 2 for each group. (**a**) “talking to your baby,” (**b**) “communicating through touch,” (**c**) “noticing” in MAIA, and (**d**) “not distracting” in MAIA. The error bars indicate standard errors. **p < .01, *p < .05, ^†^*p* < .10.

During diaper changing, interactions between the group and session were significant for the following factors “talking to your baby” and “communicating through touch” (*F* (2,126) = 4.13, *p* = 0.018, *η*^*2*^ = 0.062; *F* (2,126) = 4.46, *p* = 0.013, *η*^*2*^ = 0.066). A simple main effect of the session was significant in the IDS group when “talking to your baby,” suggesting that these subscale scores were significantly higher during Session 2 than Session 1 (*F* (1,126) = 7.24, *p* = 0.008, *η*^*2*^ = 0.225). The simple main effects of the session were significant when “communicating through touch” in both the control group and no-IDS group, suggesting that these subscale scores decreased significantly during Session 2 compared with Session 1 (*F* (1,126) = 9.21, *p* = 0.003, *η*^*2*^ = 0.107; *F* (1,126) = 5.14, *p* = 0.025, *η*^*2*^ = 0.176; Fig. 1a, b). Other maternal behavior did not change significantly (refer to the descriptive data in Supplementary Table 1). Psychological variables, Edinburgh Postnatal Depression Scale (EPDS), Perceived Stress Scale (PSS), Parenting Self-Efficacy (PSE), and State-Trait Anxiety Inventory Trait (STAI-T), had no significant effects (refer to the descriptive data in Supplementary Table 2). The MAIA (Multidimensional Assessment of Interoceptive Awareness) “noticing” and “not distracting” subscales indicated the significant main effects of the session (*F* (1, 126) = 10.514, *p* = 0.002, *η*^*2*^ = 0.077; *F* (1, 126) = 14.096, *p* < 0.001, *η*^*2*^ = 0.101). Holm-adjusted multiple comparisons revealed that the control group and the no-IDS group presented significant increases in “noticing” during Session 2 compared with Session 1 (*t* (126) =  − 2.129, *p* = 0.035; *t* (126) =  − 2.002, *p* = 0.047). In the no-IDS and IDS groups, “not distracting” also decreased significantly during Session 2 compared with Session 1 (*t* (126) =  − 2.939, *p* = 0.004; *t* (126) =  − 2.203, *p* = 0.029; Fig. 1c, d. Other MAIA subscales indicated no significant effects (refer to the descriptive data in Supplementary Table 3). The internal consistency of each psychological scale and MAIA of Sessions 1 and 2 was measured using Cronbach’s α coefficient. The values were high for the EPDS, PSS, STAI-T, and MAIA scales, that is, between 0.847 and 0.936. Additionally, the α coefficient for the PSE of Sessions 1 and 2 were 0.719 and 0.708, which indicates a satisfactory level of scale homogeneity (refer to the descriptive data in Supplementary Table 4).

### Association with interoceptive sensibility and psychological scales

**Figure 2:**
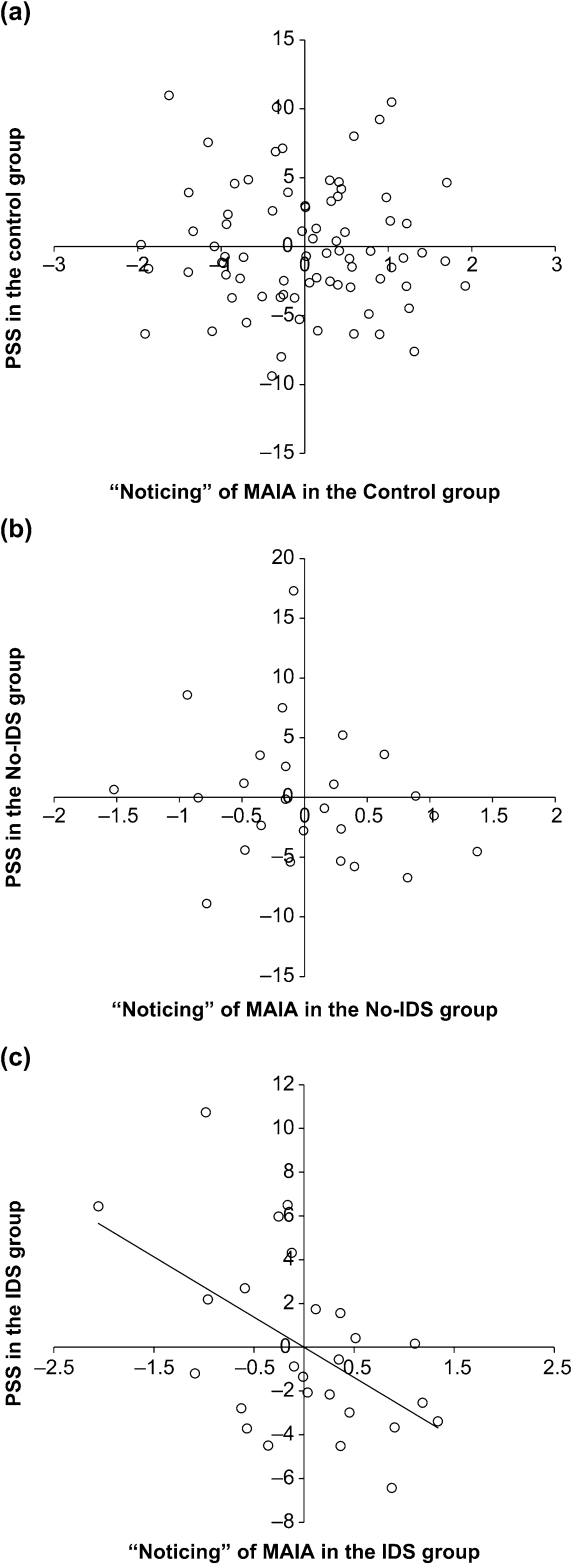
Association between changes in “noticing” and PSS for each group. (**a**) Control group (*n* = 78), (**b**) IDS group (*N* = 25), and (**c**) No-IDS group (*N* = 26). The control variables were “noticing” and PSS in Session 1. The line is the regression line.

The MAIA subscales of “noticing” and “not distracting” changed significantly in the two sessions. A partial correlation analysis of changes in these factors and changes in psychological variables (controlling for baseline i.e., first session scores) revealed that the relationship between change scores (Session 2 and Session 1) of “noticing” and PSS was significant in the IDS group only (control group, *r* (76) =  − 0.025, *p* = 0.831; no-IDS group, *r* (23) =  − 0.192, *p* = 0.380; IDS group, *r* (24) =  − 0.525, *p* = 0.008; Fig. 2a–c), suggesting that the more “noticing” increased, the more stress decreased in the IDS group. The changes in “not distracting” scores had no significant correlations with any psychological scales in all groups.

### Effects of the frequency of application usage on interoceptive sensibility

**Figure 3:**
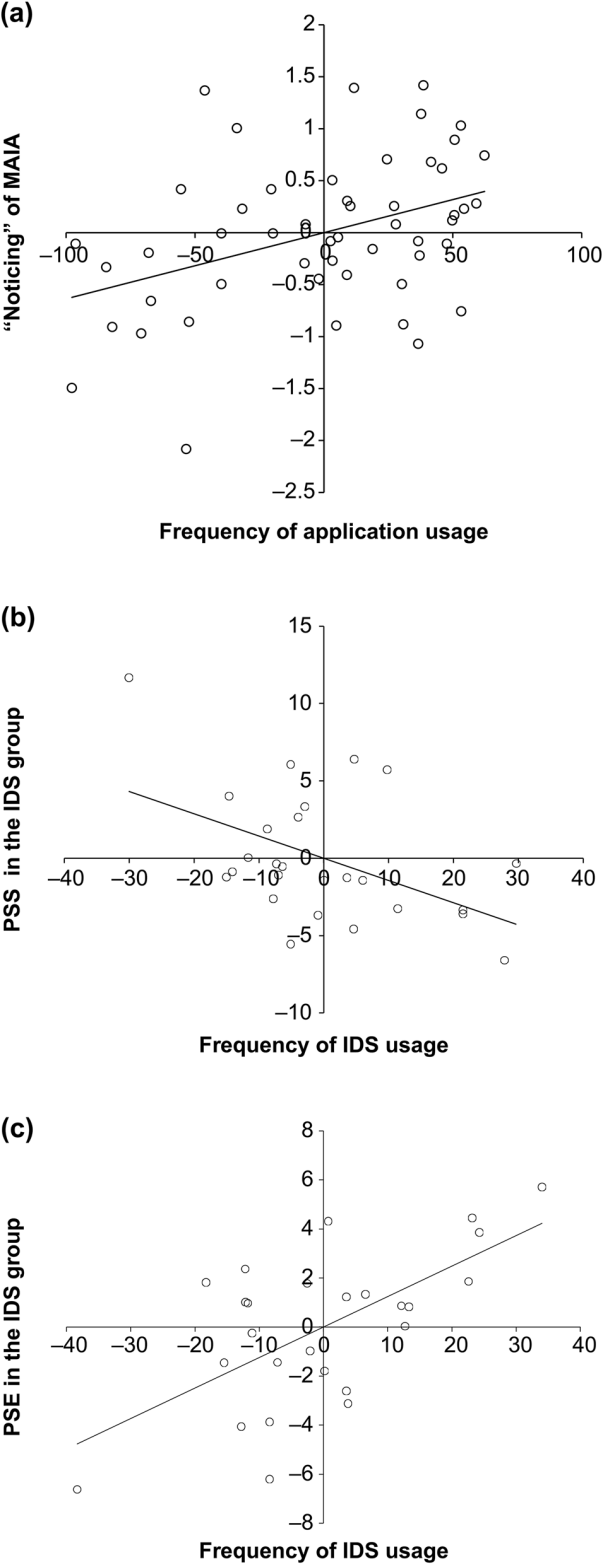
Association between interoceptive sensibility and psychological variables and the frequency of application and IDS usage. (**a**) Associations between changes in “noticing” and the frequency of application usage for the group that used the application (*N* = 51), (**b**) associations between PSS and the frequency of IDS usage in the IDS group (*N* = 26), (**c**) associations between PSE and the frequency of IDS usage in the IDS group (*N* = 26). The lines are regression lines. The scores shown are regression residuals, which control for the baseline variation in “noticing” during Session 1 and are distributed around a mean value of 0.

In the two groups that used the ESM application, a significant positive partial correlation was found between the frequency of application usage and changes in “noticing” (*r* (49) = 0.398, *p* = 0.004; Fig. 3a).

### Multilevel correlation between the use of the song and daily feelings

Table 1 presents the result of the within-level multilevel correlation between the dummy variables with or without the song (− 1 vs. 1), and the daily feelings of mothers and infants and mothers’ HR in the IDS group. There were significant correlations between the dummy variables of the song and mothers’ HR, calmness, and vitality (*r* = 0.061, *p* = 0.005; *r* =  − 0.058, *p* = 0.007; *r* = 0.103, *p* < 0.001, respectively). For infants, there were significant correlations between the dummy variables of the song and infants’ calmness, valence, and vitality (*r* =  − 0.057, *p* = 0.008; *r* = 0.052, *p* = 0.017; *r* = 0.065, *p* = 0.003, respectively). These results indicate that the mothers’ HR and vitality were higher, that is, they were more excited when the song was used than when it was not. Meanwhile, when the song was used, the infants turned to be more excited and pleasant and displayed better mood than when the song was not played.

**Table 1:** Within-level multilevel correlation coefficients between centered dummy variables with or without the song (− 1; no song, 1; with song) and mothers’ HR and mothers’ and infants’ daily feelings when the IDS group changed diapers (*N* = 2138).

	1		2		3		4		5		6		7		8		9		10	
1.The dummy variables	**0.154**	**																		
2.Mothers’ HR	0.061	**	**0.298**	**																
3.Mothers’ calmness	−0.058	**	−0.108	**	**0.324**	**														
4.Mothers’ valence	0.020		−0.054	*	0.405	**	**0.394**	**												
5.Mothers’ relaxation	0.010		−0.043	*	0.451	**	0.661	**	**0.355**	**										
6.Mothers’ vitality	0.103	**	−0.013		0.278	**	0.415	**	0.497	**	**0.324**	**								
7.Infants’ calmness	−0.057	**	−0.022		0.312	**	0.189	**	0.273	**	0.156	**	**0.283**	**						
8.Infants’ valence	0.052	*	0.010		0.241	**	0.313	**	0.320	**	0.226	**	0.518	**	**0.348**	**				
9.Infants’ relaxation	0.011		−0.022		0.263	**	0.289	**	0.335	**	0.218	**	0.600	**	0.825	**	**0.316**	**		
10.Infants’ vitality	0.065	**	−0.028		0.192	**	0.262	**	0.292	**	0.222	**	0.431	**	0.795	**	0.788	**	**0.256**	**

Intra-class correlations are on the diagonal and in bold.

**p < .01, *p < .05.

### Effect of the frequency of IDS usage on psychological scales in the IDS group

In the IDS group, a partial correlation analysis of the frequency of IDS usage and changes in the psychological variables revealed that the relationship between the frequency of IDS usage and change scores of PSS and PSE was significant (*r* (24) =  − 0.479, *p* = 0.014 and *r* (24) = 0.630, *p* = 0.001; Fig. 3b, c), suggesting that the greater the frequency of IDS usage, the more PSS decreases and the more PSE increases.

### The effects of mothers’ HR and interoceptive sensibility on mothers’ and infants’ daily feelings and the mental health of mothers

**Figure 4:**
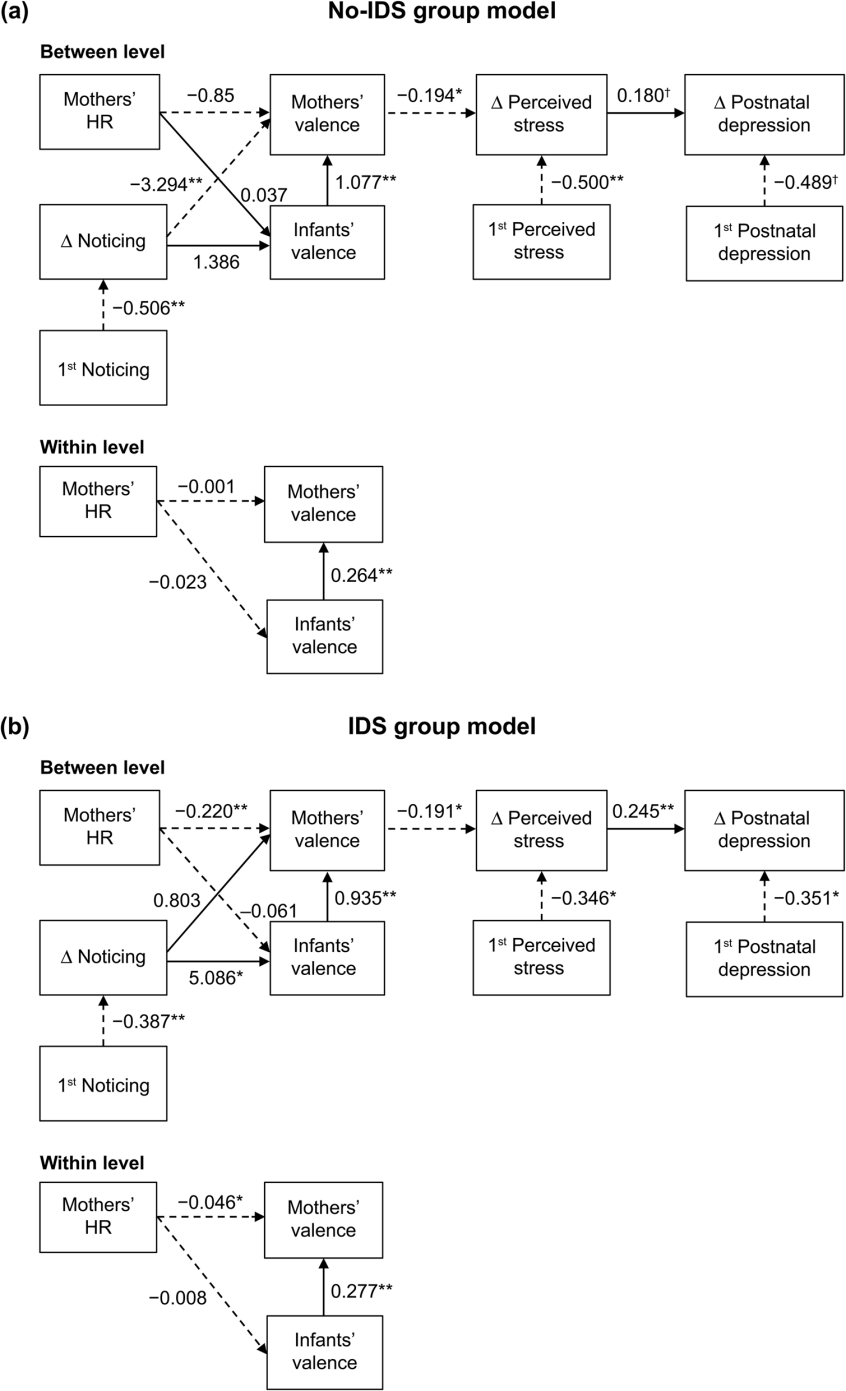
Multiple group multilevel structural equation model. (**a**) No-IDS group model, (**b**) IDS group model. Solid line: Positive effect, Broken line: Negative effect. Estimation: Maximum likelihood estimation, the path of notation unstandardized coefficients. Infants’ valence was evaluated by their mothers. Δ indicates the change score (Session 2 minus Session 1). **p < .01, *p < .05, ^†^*p* < .10.

The results of the multiple group multilevel structural equation modeling (SEM) analysis in the no-IDS and IDS groups are shown in Fig. 4. According to this model, the result of the χ^2^ test was not significant (χ^2^ (40) = 53.101, *p* = 0.080), with comparative fit index (CFI) = 0.976 and root mean square error of approximation (RMSEA) = 0.011, which is considered a good fit; thus, it seemed that the model fit the data. In the no-IDS group, the increase in MAIA “noticing” scores affected the mothers’ valence. A significant difference was found between the no-IDS group and the IDS group in the path coefficient of changes in “noticing” to the mothers’ valence (*p* = 0.003). Meanwhile, in the IDS group, the increase in MAIA “noticing” scores increased the infants’ valence, as evaluated by their mothers. The mothers’ valence significantly reduced their perceived stress and postnatal depression. Details of all pass coefficients are listed in Supplementary Table 5.

A previous study has reported that the facets of interoceptive sensibility and STAI-T were associated through mediation by the appraisal of one’s own body as safe and trustworthy^38^. We conducted an a posteriori explanatory analysis to examine this issue. The result verified that in the no-IDS group, but not in the IDS group, the awareness of body sensation (noticing) was linked with less pleasant feelings via the mediation of the belief of body trusting (Supplementary Fig. 1).

## Discussion

The results of this study confirmed that the IDS intervention with social touch was associated with improved mother–infant interactions to a certain extent. The mothers in the IDS group increased “talking to your baby” during diaper changes. Consistent with a previous study^39^, maternal affectionate and stimulating touch decreased as infants got older, therefore “communicating through touch” decreased in the control and no-IDS groups; however, the level of this type of interaction was maintained in the IDS group. The multilevel correlation analysis showed that mothers in the IDS group had higher levels of subjective arousal, vitality, and higher HRs after using the song during diaper changing. Likewise, in the IDS group, infants’ level of arousal, valence, and good mood as assessed by mothers were higher when using the song. Given that the feelings of the infants were assessed by the mothers, it is possible that infants’ feelings may be similar to those of the mothers by their social projection (projecting their own internal state to interpret the reactions of their infants)^40^. The frequency of IDS usage was associated with lower perceived stress and higher parenting self-efficacy. These results validate that IDS with social touch possibly created experiences of shared enjoyment between the mothers and the infants and promoted positive interactions. Furthermore, it was suggested that the enhancement of the mother–infant interactions mediated by IDS is effective for increasing parenting self-efficacy and reducing the perceived stress of mothers.

Both in the IDS and no-IDS groups, MAIA “noticing” subscale scores increased and the “not distracting” scores decreased between Session 1 and Session 2. The “noticing” score increased even in the control group, which did not experience any interventions. Parenting infants and paying attention to their physical states require the constant activation of the interoceptive network in the parents’ brain^31^; thus, a mother’s subjective body awareness may be naturally increased through parenting experiences. However, the increase of “noticing” scores was significantly associated with the reduction of perceived stress levels in the IDS group but not in the no-IDS or control groups. In addition, HR measurement frequencies correlated with increased interoceptive sensibility (noticing) in the IDS and no-IDS groups that used the application. These results suggest the utility of daily HR measurements as itself an intervention that enhances interoceptive awareness, and it may have improved the well-being of mothers during parenting in the IDS group.

The results of the multiple group multilevel SEM indicate that increased interoceptive sensibility (noticing) was related to the enhanced observation of the positive feelings of the infants in the IDS group. In the IDS group, mothers and infants repeated their experiences of noticing infants’ positive feelings through the infants’ facial expressions and voices, with physical contact. Through these experiences of mother–infant interaction, mothers who became more aware of their interoceptive sensibility reported enhanced awareness of infants’ positive feelings. Interoception is not only important during the processing of emotional experiences and self-regulation but is also associated with social cognitive abilities, such as the ability to understand others’ emotions and empathy^24–26,41^. Based on a previous finding that direct gaze increases interoceptive accuracy, we infer that the increased opportunities to gaze at each other increased the mothers’ interoceptive sensibility^42,43^. However, gaze is not the only source of influence, and other interactions (touch, sound etc.) may have affected it.

In the no-IDS group, an increase in interoceptive sensibility (noticing) was negatively associated with mothers’ positive feelings. Recent studies have reported that interoceptive sensibility, measured by scales such as MAIA, is a powerful predictor of anxiety, emotional discrimination, and emotional regulation^28,29,44,45^. During routine parenting, infants communicate hunger and discomfort to their caregivers by signals, such as crying and groaning, to adjust their homeostasis. It could be inferred that mothers’ perceptions of their demand signals do not enhance mothers’ positive feelings. When changing diapers in the no-IDS group, there was no active intervention to evoke positive feelings in mothers and infants; therefore, as it involved a hygiene task, the mothers’ attention may have been directed to diapers rather than the infants’ facial expressions and gazes. Therefore, the mothers’ increased interoceptive sensibility appeared to be directed toward their own feelings.

These interpretations can be partially supported by the overall higher levels of anxiety of the mothers in this study, which were indicated by the above 40 mean STAI-T scores in all groups^46,47^.This might be because of the difficult and stressful situations of mothers with infants in the social and cultural context of Japan^48^. The result of the a posteriori explanatory analysis verified that in the no-IDS group only, the awareness of body sensation (noticing) was associated with less pleasant feelings and the belief of body trusting mediated this relationship. These results demonstrated that high levels of attention toward internal sensations (noticing) accompanied by increased trust into one's own body sensations are associated with less pleasant feelings. Hence, the discrepancy between the prediction about the body generated by the belief of the healthy body and focused stress-related signals from the actual body (prediction error) might cause anxiety-related negative feelings^34,49^. The intervention with IDS and social touch adopted in this study might also buffer the negative effect of interoceptive sensibility and promote the awareness of infants’ positive emotions, as a result of the positive mother–infant experience. The frequency-dependent effects of IDS reported above further support the following interpretation: Mothers who use IDS more frequently report higher self-efficacy and less stress.

The activation of the anterior insula, which is a critical brain region for interoception, has been linked previously with sensitive parenting behaviors and qualified parent–child interaction^31^. Mothers’ interoceptive knowledge has been associated with school-aged children’s emotional regulation and social affective skills^32^. Improving caregivers’ interoception and social cognition during parenting may lead to infants’ enhanced interoception and social development. The current study partially identified how maternal interoceptive sensibility relates to the daily feelings of mothers and infants during parenting and their well-being when using the IDS intervention. These findings may help develop appropriate techniques for enhancing mother–infant interactions.

Some limitations of this study should be recognized. First, this study could not distinguish whether IDS sounds and rhythms enhanced positive emotions, or whether IDS-promoted mother–infant interactions enhanced positive emotions. Second, the dimension of interoception was examined using the subjective interoceptive sensibility index^27^ rather than psychophysiological assessments, such as the heartbeat detection task^50^. We tried a new method, which included a heartbeat detection task using a smartphone, but the data could not be examined due to technical problems. Future studies need to examine how the interoceptive accuracy of caregivers affects daily mother–infant interactions by improving the methods used. Third, we have to recognize the potential for a placebo effect. As participants actively measured their physiological indices and responded to the questionnaire using a smartphone application, participants in the IDS group especially could infer the aims of this study, and this may have influenced the results consciously and unconsciously. Future studies could overcome this limitation by using wearable devices (HR measurements and accelerometer postural measurements) that can be automatically uploaded to the investigator’s database.

Despite these limitations, this study appears to be the first trial to suggest that maternal interoceptive sensibility mediates the relationship between IDS effects and social touch and maternal and infant well-being under controlled experimental conditions. Through the experience of affective interactions between mothers and infants, the increased subjective bodily awareness of mothers also increased their awareness of their infants’ positive feelings—possibly creating a positive feedback loop. In contrast, self-awareness in the absence of a programmed positive mother–infant interaction led to a negative association between maternal self-awareness and the mood state of the mother and infant in the no-IDS group. Together, these findings raise the possibility that a simple behavioral intervention could deter or alleviate clinical syndromes, such as post-partum depression, by enhancing the quality of mother–infant interactions. The findings of this study also provide a basis for investigating the role of maternal interoception in promoting mothers’ and infants’ well-being and cognitive and behavioral developments (e.g., stress self-regulation) in prospective longitudinal studies.

## Methods

### Study design

A two-factor mixed design was used: group (between-participant factor: control, no-IDS, and IDS) × session (within-participant factor: Session 1 and Session 2). Mothers’ psychological characteristics were measured by a web survey at Sessions 1 (pre-questionnaire) and Session 2 (post-questionnaire that was one month later from Session 1). Mothers’ and infants’ behaviors during diaper changes were also assessed. For a month between Sessions 1 and 2, mothers in the no-IDS and IDS groups measured their own and their infants’ daily feelings and their own HR with a smartphone application using the ESM, one to three times a day. The IDS group was asked to play and sing a song to facilitate mother–infant interaction one to three times a day. The control group was not instructed to follow anything other than their usual routine between Sessions 1 and 2. They were also not informed of activities of other groups. One month after administering the Session 1 questionnaire, the Session 2 questionnaire was given to all participants. The response period was one week, and subjects who did not respond within the period were excluded. The entire process of the experiment was conducted online using email and websites, and there was no direct, face-to-face contact with the participants.

### Participants

**Figure 5:**
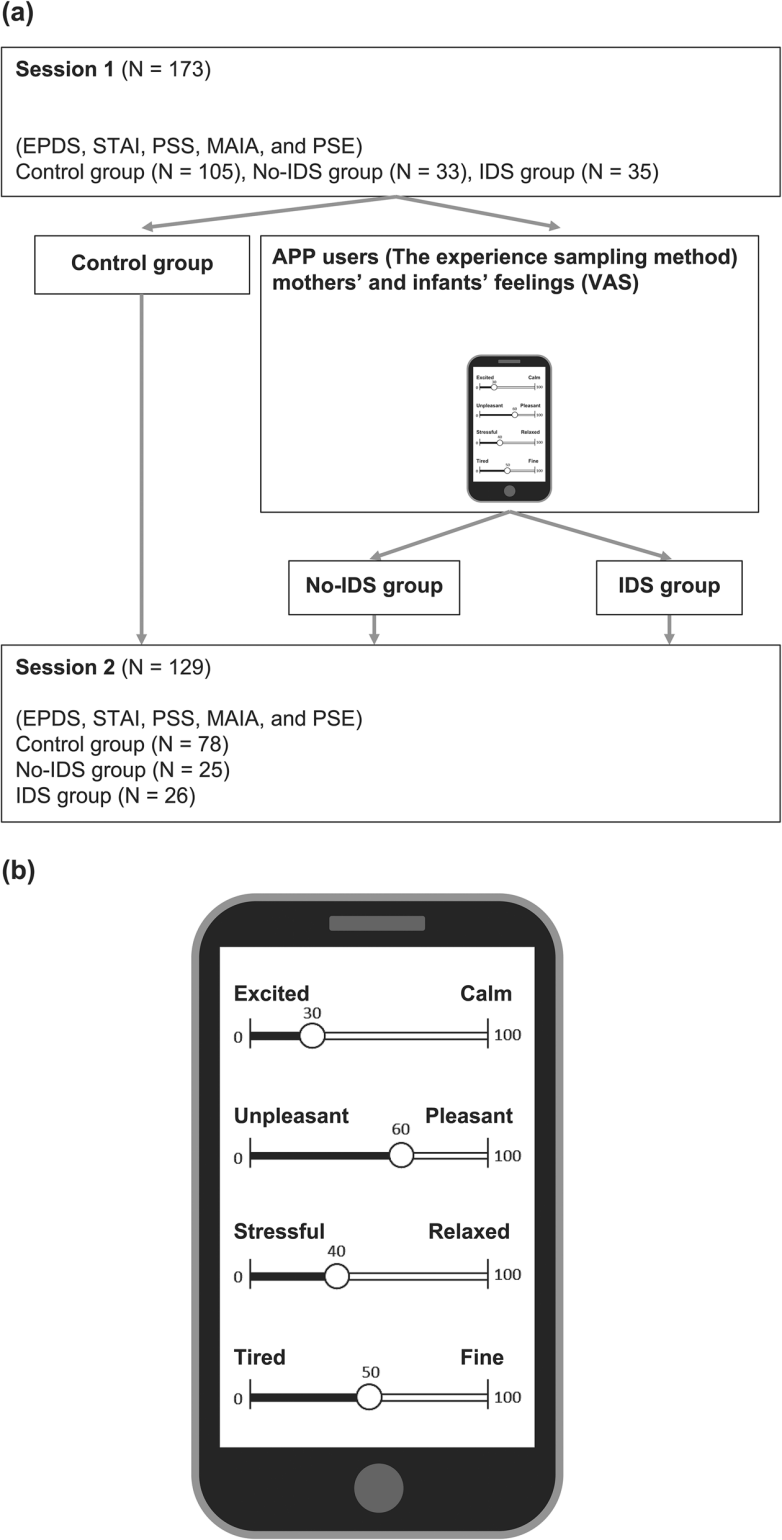
Explanation of the research flow and the smartphone application screen. (**a**) Research flow chart and (**b**) VAS answer screen about mothers’ and infants’ feelings.

The participants were recruited among the users of a Japanese caregiving diary application (First Ascent inc.). One hundred and seventy-three pairs of healthy mothers and their first-born infants (2–8 months) who reported no diagnosis of mental illness or cardiovascular diseases participated in Session 1. One hundred and twenty-nine mothers (mean age = 31.3 years, standard deviation (SD) = 5.17) and their infants (male: 67, mean age = 4.55 months, SD = 1.79) who answered appropriately during Sessions 1 and 2 were included in the analysis. The excluded cases were 20 mothers who did not participate in the second session, 21 mothers who did not appropriately answer the instructional manipulation check (IMC)^51^, and 3 mothers who used the application less than 11 times. Details are described in the Supplementary Information file. The number of participants in each group was 78 in the control group, 25 in the no-IDS group, and 26 in the IDS group. There were no significant differences between the groups according to the age (months), gender of the infants, and the age of the mothers (Table 2). The research flowchart is shown in Fig. 5a. The process used to determine the participants is shown in the Supplementary Information file.

**Table 2:** Basic characteristics of the participants.

	*N*	Control group	No-IDS group	IDS group	Total	Main effect	
		78	25	26	129	(F-Value)	(*P*-Value)
Infant	Male	40	11	16	67	0.793	.455
	Female	38	14	10	62		
	Age (months)	2–8	2–8	2–8	2–8	0.881	.417
	Mean	4.718	4.240	4.346	4.550		
	SD	1.712	1.985	1.853	1.794		
Mother	Age (years)	20–44	24–42	23–41	20–44	2.307	.104
	Mean	30.513	32.240	32.692	31.287		
	SD	5.207	5.286	4.654	5.169

### Instruments

Postnatal depression was assessed using the EPDS^52^. It consists of 10 items on a 4-point scale, with higher scores indicating greater severity of depression. The participants were all healthy volunteers, so the use of the scale was intended to target this specific part of a woman’s life (i.e., the postnatal period), and the scores were expected to reflect mood variation in response to experimental manipulation rather than clinical depression. Anxiety was assessed using STAI^53,54^, which comprises two factors: (1) the anxiety levels at the present moment (20 items) and (2) the anxiety levels as a personal characteristic (20 items). Interoceptive sensibility was assessed using the MAIA, which consists of 32 items measured on a 6-point scale^55^. The MAIA consists of 8 subscales: noticing, not distracting, not worrying, attention regulation, emotional awareness, self-regulation, body listening, and trusting. Higher scores indicate greater interoceptive sensibility. Perceived stress was assessed using PSS-10, which consists of 10 items measured on a 5-point scale^56–58^. Higher scores indicate greater perceived stress. PSE was assessed using the PSE Scale, which consists of 13 items measured on a 5-point scale^59^. PSE refers to the degree of confidence that mothers with infants have in their ability to flexibly manage the challenges of parenting^60^. The higher the score, the greater the PSE. To assess the behavioral exchanges between the mothers and infants during diaper changes, the mothers assessed their own behaviors as well as those of their infants according to the five items on a 5-point scale. These behavioral items were chosen with reference to previous studies for typical behaviors during diaper changes^61,62^. In this case, the higher the score, the more frequent the behaviors. The items included “You talk to your baby,” “You are smiling,” “Your baby is smiling,” “Your baby is crying,” and “You communicate with your baby through touch.”

The application used by the no-IDS and IDS groups had common functions as follows: daily feelings recording, HR measurement, and the heartbeat-counting task. Only the IDS group used the application with an infant-directed song during diaper changes. Summary statistics on the frequency of application and IDS usage are described in Table 3. There was no significant difference between the no-IDS and IDS group in the frequency of the application usage and diaper changing. The mean frequency of IDS usage was 59.46 (SD = 29.98).

**Table 3:** Summary statistics on frequency of application usage and IDS usage.

		Mean	Median	SD	Min	Max	*t-value*	df	*p-value*
Frequency of application usage	No-IDS group	101.000	113.000	48.743	12.000	160.000	− 1.065	49	.292
	IDS group	114.769	120.500	43.530	36.000	167.000			
Frequency of diaper changing	No-IDS group	70.760	80.000	30.422	7.000	110.000	− 1.426	49	.160
	IDS group	83.231	82.500	31.963	27.000	150.000			
Frequency of IDS usage	IDS group	59.462	53.000	29.980	12.000	148.000			

### Infant-directed song during diaper changes (IDS)

The IDS was originally produced (lyrics, music, and song by “Tomoka Fujioka”; Supplementary Table 6), and was easily playable video on the application. In the video clip, the infants and mothers were changing diapers while interacting with each other. The mothers replayed the video and used the audio portion to generate the song as a musical backdrop to their interactive play while changing diapers. The length of the IDS was about 50 s, and the IDS had lyrics that encouraged physical interaction between mothers and infants during diaper changes, such as “rub your round belly” and “squeeze soft cheeks.” In the current study, social touch was defined as an affectionate touch (e.g., gently stroking, kissing, and hugging) and stimulating touch (e.g., tickling and poking) caused by the lyrics of the song^39,63^. The mothers were instructed to see the detailed and illustrated instructions from the application (not only videos) and conduct the tasks.

### Daily feelings recording

The mothers’ and infants’ daily feelings were recorded by the mothers using a visual analog scale (VAS) on the application. Since entering the time of day was not instructed to the mothers, they chose the time of day on their own during caregiving. When the mothers changed diapers, they were instructed to answer after doing so. To see if they changed diapers, when entering the daily feeling data, the mothers answered whether they had changed diapers and whether they used IDS in the IDS group only. Diaper changing and IDS intervention were confirmed from the data entered in the application. The instructions to the mothers were, “Please describe how you are feeling right now” and “Please mention you baby’s state.” The mothers answered by inferring their infants’ feelings from their current state. There were four VAS items: calmness, excited (0)–calm (100); valence, unpleasant (0)–pleasant (100); relaxation, stressful (0)–relaxed (100); and vitality, tired (0)–fine (100). Meanwhile, for infants, vitality was assessed on a scale of bad mood (0)–good mood (100). The response screen on the app is shown in Fig. 5b.

### HR measurement using smartphone photoplethysmography

The application used by the no-IDS and IDS groups included a function to measure HR based on changes in blood flow luminance by placing a fingertip on a smartphone camera for approximately 30 s. This function used the SDK (software development kit produced by WIN Frontier Co,. Ltd.) whose reliability was reported^64^. Mothers in the no-IDS and IDS groups measured their own and their infants’ HRs on the application after entering the daily feelings data. The measured HR was reported on the application. The mother measured her HR while sitting down. The infants’ HRs were measured by their mothers, while the infants were asleep. With reference to previous research^65^, we set a cut-off value of 100 bpm to detect whether the infants’ HRs were properly measured in this study. A total of 2,108 infant HR data specimens were obtained on the application. Of these infant HR data, 18.4% were below 100 bpm. Therefore, infants’ HRs were not accurately measured in this study and were excluded in the analysis.

### Heartbeat-counting task

To measure maternal interoceptive accuracy, the application also included a heartbeat-counting task where the mothers were asked to count their own heartbeats within a certain period. The heartbeat-counting task on the application (HCT-A) was performed three times (on the 1st, 14th, and 28th day) at home. To verify the reliability and validity of HCT-A, we compared the HCT-A scores with Schandry’s heartbeat-counting task using an electrocardiogram^50^ at the laboratory. The reliability and validity of the HCT-A were considered adequate. Details of the methods and results of the comparison are described in Supplementary Figs. 2, 3. Unfortunately, many mothers failed to perform the heartbeat measurement appropriately; therefore, maternal interoceptive accuracy obtained from the HCT-A was excluded in the analysis.

### Instruction

How to use the application and perform the various tasks were taught through videos wherein the models were filmed operating the application and performing the tasks, after which explanatory texts were displayed as subtitles. Besides the video, an illustrated instruction manual was produced and the participants were instructed to view it before starting the examination from the website and application.

### Statistical analysis

A two-factor analysis of variance (ANOVA) was conducted to examine how mothers’ and infants’ behaviors during diaper changes, maternal psychological scales, and maternal interoceptive sensibility changed from Session 1 to Session 2 in each group. The explanatory variables were group (control group, no-IDS group, and IDS group) and session (Sessions 1 and 2). The objective variables were the mothers’ and infants’ behaviors during diaper changes, maternal psychological scales (EPDS, PSS, STAI-T, and PSE), and eight MAIA subscales.

To examine the relationship between the changes in the eight MAIA subscales and changes in maternal psychological scales, a partial correlation analysis was conducted in each group, controlling for baseline variations of the eight MAIA subscales and maternal psychological scales during Session 1. In the no-IDS and IDS groups, a partial correlation analysis was conducted to confirm the association between the frequency of application usage and changes in maternal interoceptive sensibility controlling for baseline maternal interoceptive sensibility during Session 1. The change in each index was calculated by subtracting the score from Session 1 with the score from Session 2.

To confirm whether there were significant influences of the group on individuals’ responses, a multilevel analysis was needed; therefore, we calculated the intra-class correlation coefficients (ICC) to see if mothers’ and infants’ daily feelings and the maternal HR obtained using the ESM had a hierarchical structure. The ICC for each scale ranged from .219 to .392 (Supplementary Table 7), suggesting that mothers’ and infants’ daily feelings and maternal HR had a hierarchical structure^66^, and multilevel analyses were performed. To examine the association between the IDS use and mothers’ and infants’ daily feelings in the IDS group, we conducted a multilevel correlation analysis (within-level) of the daily feelings of mothers and infants and mothers’ HR with IDS use using centered dummy variables (no song =  − 1, with song = 1). Pair-wise deletion was used to treat the incomplete data in the selection.

In the IDS group, a partial correlation analysis was conducted to confirm the association between the frequencies of IDS usage and changes in the psychological scales, control for the baseline in each psychological variation, and determine the frequency of the application usage and diaper changes.

We conducted multiple group multilevel SEM with the data collected from the no-IDS and IDS groups to explore the causal structures of the associations between the IDS and changes in mothers’ and infants’ daily feelings, maternal HR, maternal psychological variables, and maternal interoceptive sensibility (noticing). In the between-levels analysis, mothers’ and infants’ daily feelings, maternal HR, Session 1 scores, and changes in each interoceptive sensibility scale, perceived stress, and postnatal depression were included. Changes in “noticing,” perceived stress, and postnatal depression were controlled by partialling out Session 1 scores for each scale. Mothers’ and infants’ daily feelings and maternal HR were included in the within-levels analysis. The robust maximum likelihood method was used for estimation. The ideal fit of the structural model was evaluated using standard indices such as RMSEA and CFI.

HAD17.0^67^ was used to conduct the two-factor ANOVA, correlation analysis, and multilevel analysis. Mplus version 8.4^68^ was used to perform the multiple group SEM.

### Ethical statement

This study was conducted in compliance with the Declaration of Helsinki, and all experimental protocols were approved by the Ethics Committee of Nagoya University (approval number: NUPSY-190510-G-01) and pre-registered (UMIN study ID: UMIN000036708, enrollment date: May 15, 2019). In addition, signed informed consent was obtained from all participants before the study.

## Supplementary Information

Supplementary Information.

Supplementary Figure 1.

Supplementary Figure 2.

Supplementary Figure 3.

## Data Availability

All data analyzed during this study are included in this published article (and its Supplementary Information files).
