# Supplementary material for: Mothers’ interoceptive sensibility mediates affective interaction between mother and infant
Source: Sci Rep. 2022 Apr 15;12:6273. doi: 10.1038/s41598-022-09988-y (PMC9011379; doi:10.1038/s41598-022-09988-y)
Supplement: Supplementary file 1 — Supplementary Information. [file 41598_2022_9988_MOESM1_ESM.docx]

**Supplementary Information for Manuscript: Mothers’ Interoceptive Sensibility Mediates Affective Interaction between Mother and Infant**

Ayami Suga^1, 2*^, Yosuke Naruto^1^, Venie Viktoria Rondang Maulina^2, 3^, Maki Uraguchi^2^, Yuka Ozaki^4^, Hideki Ohira^2^

^1^Unicharm Corporation, 769-1602 Kagawa, Japan

^2^Department of Psychology, Nagoya University, 464-8601 Nagoya, Japan

^3^Department of Psychology, Atma Jaya Catholic University of Indonesia, Jakarta, Indonesia

^4^Department of Social Psychology, Toyo University, 112-8606 Tokyo, Japan

*ayami-suga@unicharm.com

**Supplementary Table 1. Summary statistics and ANOVA results of behaviors during diaper change by group and session**

***p* < .01, **p* < .05, ^†^ *p* < .10

|  | Group | Control (n = 78) | | No-IDS (n = 25) | | IDS (n = 26) | | Main effect (*F*-Value) | |  |
| --- | --- | --- | --- | --- | --- | --- | --- | --- | --- | --- |
|  | Session | Session 1 | Session 2 | Session 1 | Session 2 | Session 1 | Session 2 | Group | Session | Interaction |
| You talk to your baby | *mean* | 4.538 | 4.667 | 4.680 | 4.480 | 4.269 | 4.654 | 0.465 | 2.020 | 4.130^*^ |
|  | *SD* | 0.768 | 0.733 | 0.557 | 0.586 | 1.079 | 0.562 |  |  |  |
| You are smiling. | *mean* | 4.167 | 4.077 | 4.400 | 4.040 | 3.885 | 4.077 | 0.608 | 0.932 | 2.498^†^ |
|  | *SD* | 0.889 | 0.894 | 0.577 | 0.978 | 1.143 | 0.845 |  |  |  |
| Your baby is smiling | *mean* | 3.667 | 3.513 | 3.600 | 3.480 | 3.308 | 3.385 | 0.648 | 0.398 | 0.491 |
|  | *SD* | 1.028 | 1.137 | 0.957 | 1.122 | 1.123 | 1.061 |  |  |  |
| Your baby is crying. | *mean* | 2.526 | 2.474 | 2.560 | 2.240 | 2.577 | 2.346 | 0.134 | 3.130^†^ | 0.646 |
|  | *SD* | 0.990 | 1.090 | 1.083 | 0.879 | 0.987 | 0.977 |  |  |  |
| You communicate with your baby through touch. | *mean* | 4.154 | 3.821 | 4.040 | 3.600 | 3.731 | 4.000 | 0.450 | 2.957^†^ | 4.469^*^ |
|  | *SD* | 0.898 | 1.066 | 0.841 | 1.225 | 1.116 | 0.693 |  |  |  |

**Supplementary Table 2. Summary statistics and ANOVA results of psychological variables by group and session**

***p* < .01, **p* < .05, ^†^ *p* < .10

|  | Group | Control (n = 78) | | No-IDS (n = 25) | | IDS (n = 26) | | Main effect （*F*-Value） | |  |
| --- | --- | --- | --- | --- | --- | --- | --- | --- | --- | --- |
|  | Session | Session 1 | Session 2 | Session 1 | Session 2 | Session 1 | Session 2 | Group | Session | Interaction |
| EPDS | *mean* | 5.467 | 5.936 | 3.800 | 3.500 | 4.654 | 5.423 | 2.284 | 0.542 | 0.610 |
|  | *SD* | 4.428 | 4.746 | 3.069 | 3.217 | 4.195 | 4.365 |  |  |  |
| PSS | *mean* | 17.808 | 17.397 | 15.680 | 15.200 | 17.615 | 16.846 | 1.156 | 1.107 | 0.046 |
|  | *SD* | 6.878 | 6.842 | 6.101 | 6.752 | 6.986 | 6.214 |  |  |  |
| PSE | *mean* | 49.727 | 49.821 | 51.440 | 51.875 | 51.115 | 50.577 | 1.101 | 0.001 | 0.283 |
|  | *SD* | 6.506 | 6.049 | 5.973 | 7.110 | 5.922 | 5.240 |  |  |  |
| STAI-T | *mean* | 42.410 | 42.026 | 37.920 | 39.960 | 40.731 | 40.538 | 1.158 | 0.592 | 1.445 |
|  | *SD* | 10.428 | 10.277 | 8.281 | 11.631 | 9.726 | 9.517 |  |  |  |
| STAI-S | *mean* | 36.577 | 36.141 | 33.320 | 34.080 | 35.654 | 35.731 | 0.991 | 0.028 | 0.221 |
|  | *SD* | 9.571 | 9.248 | 7.175 | 10.299 | 7.792 | 9.168 |  |  |  |

EPDS: Edinburgh Postnatal Depression Scale; PSS: Perceived Stress Scale; PSE: Parenting Self-Efficacy; STAI-T: State-Trait Anxiety Inventory Trait; STAI-S: State-Trait Anxiety Inventory State.

**Supplementary Table 3. Summary statistics and ANOVA results of MAIA subscales by group and session**

***p* < .01, **p* < .05, ^†^ *p* < .10

|  | Group | Control (n = 78) | | No-IDS (n = 25) | | IDS (n = 26) | | Main effect (*F*-Value) | |  |
| --- | --- | --- | --- | --- | --- | --- | --- | --- | --- | --- |
|  | Session | Session 1 | Session 2 | Session 1 | Session 2 | Session 1 | Session 2 | Group | Session | Interaction |
| Noticing | *mean* | 1.907 | 2.154 | 1.880 | 2.290 | 2.029 | 2.375 | 0.412 | 10.514^**^ | 0.276 |
|  | *SD* | 1.021 | 1.006 | 0.982 | 0.825 | 0.906 | 0.978 |  |  |  |
| Not Distracting | *mean* | 3.077 | 2.944 | 3.440 | 2.760 | 3.013 | 2.513 | 0.804 | 14.096^**^ | 2.547^†^ |
|  | *SD* | 1.276 | 1.166 | 1.113 | 0.965 | 1.018 | 1.205 |  |  |  |
| Not Worrying | *mean* | 2.470 | 2.462 | 2.893 | 2.840 | 2.744 | 2.474 | 1.936 | 1.518 | 0.843 |
|  | *SD* | 1.011 | 1.091 | 0.821 | 1.014 | 0.871 | 0.905 |  |  |  |
| Attention Regulation | *mean* | 2.767 | 2.650 | 2.766 | 2.766 | 2.511 | 2.401 | 0.770 | 0.673 | 0.160 |
|  | *SD* | 1.107 | 1.088 | 0.984 | 0.993 | 1.137 | 1.258 |  |  |  |
| Emotional Awareness | *mean* | 2.818 | 2.905 | 2.768 | 2.848 | 2.615 | 3.023 | 0.038 | 2.591 | 0.772 |
|  | *SD* | 1.116 | 1.146 | 1.198 | 1.117 | 1.103 | 1.178 |  |  |  |
| Self-Regulation | *mean* | 2.971 | 2.798 | 3.000 | 2.650 | 2.808 | 2.817 | 0.077 | 2.811^†^ | 0.803 |
|  | *SD* | 1.055 | 1.054 | 0.933 | 0.992 | 1.145 | 1.234 |  |  |  |
| Body Listening | *mean* | 2.500 | 2.321 | 2.187 | 2.187 | 2.179 | 2.154 | 0.693 | 0.309 | 0.289 |
|  | *SD* | 1.261 | 1.233 | 1.251 | 1.198 | 1.300 | 1.383 |  |  |  |
| Trusting | *mean* | 2.944 | 2.774 | 2.973 | 2.680 | 2.718 | 2.769 | 0.104 | 1.927 | 0.826 |
|  | *SD* | 1.299 | 1.203 | 1.045 | 1.108 | 1.355 | 1.134 |  |  |  |

**Supplementary Table 4. Internal consistency: Cronbach’s α coefficient of psychological variables and MAIA (n = 129)**

|  | Session 1 | | |  | Session 2 | | |
| --- | --- | --- | --- | --- | --- | --- | --- |
|  | α coefficient | 95% Lower | 95% Upper |  | α coefficient | 95% Lower | 95% Upper |
| EPDS | .865 | .828 | .897 |  | .863 | .825 | .896 |
| PSS | .847 | .804 | .884 |  | .854 | .814 | .889 |
| PSE | .719 | .642 | .785 |  | .708 | .628 | .777 |
| STAI-T | .902 | .876 | .925 |  | .922 | .900 | .940 |
| STAI-S | .902 | .876 | .925 |  | .923 | .903 | .941 |
| MAIA | .936 | .920 | .951 |  | .907 | .883 | .929 |

EPDS: Edinburgh Postnatal Depression Scale; PSS: Perceived Stress Scale; PSE: Parenting Self-Efficacy; STAI-T: State-Trait Anxiety Inventory Trait; STAI-S: State-Trait Anxiety Inventory State.

**Supplementary Table 5. The detail of all pass coefficients**

***p* < .01, **p* < .05, ^+^ *p* < .10

|  |  | **(a) No-IDS group** | | |  |  | **(b) IDS group** | | |  |
| --- | --- | --- | --- | --- | --- | --- | --- | --- | --- | --- |
|  |  | Estimate |  | *S.E* | *P-Value* |  | Estimate |  | *S.E* | *P-Value* |
| **Within Level** |  |  |  |  |  |  |  |  |  |  |
| Mothers’ valence on | Infants’ valence | 0.264 | ** | 0.038 | 0.000 |  | 0.277 | ** | 0.048 | 0.000 |
|  | Mothers’ HR | −0.001 |  | 0.025 | 0.953 |  | −0.046 | * | 0.022 | 0.041 |
| Infants’ valence on | Mothers’ HR | −0.023 |  | 0.037 | 0.534 |  | −0.008 |  | 0.049 | 0.864 |
| Intercepts |  |  |  |  |  |  |  |  |  |  |
|  | Mothers’ valence | 0.000 |  | 0.000 | 0.157 |  | 0.000 |  | 0.000 | 0.745 |
|  | Infants’ valence | 0.000 |  | 0.000 | 0.180 |  | 0.000 |  | 0.000 | 0.139 |
| Residual Variances |  |  |  |  |  |  |  |  |  |  |
|  | Mothers’ valence | 166.202 | ** | 34.128 | 0.000 |  | 151.864 | ** | 30.463 | 0.000 |
|  | Infants’ valence | 288.224 | ** | 41.729 | 0.000 |  | 222.959 | ** | 40.986 | 0.000 |
| **Between Level** |  |  |  |  |  |  |  |  |  |  |
| Mothers’ valence on | Infants’ valence | 1.077 | ** | 0.132 | 0.000 |  | 0.935 | ** | 0.050 | 0.000 |
|  | ΔNoticing | −3.294 | ** | 1.188 | 0.006 |  | 0.803 |  | 0.734 | 0.274 |
|  | Mothers’ HR | −0.185 |  | 0.161 | 0.250 |  | −0.220 | ** | 0.080 | 0.006 |
| ΔPerceived stress on | Mothers’ valence | −0.194 | * | 0.092 | 0.035 |  | −0.191 | * | 0.092 | 0.038 |
|  | 1^st^ Perceived stress | −0.500 | ** | 0.151 | 0.001 |  | −0.346 | ** | 0.125 | 0.006 |
| Infants’ valence on | Mothers’ HR | 0.037 |  | 0.190 | 0.845 |  | −0.061 |  | 0.229 | 0.789 |
|  | ΔNoticing | 1.386 |  | 2.235 | 0.535 |  | 5.086 | * | 2.207 | 0.021 |
| ΔPostnatal depression on | ΔPerceived stress | 0.180 | † | 0.109 | 0.098 |  | 0.245 | ** | 0.094 | 0.009 |
|  | 1^st^ Postnatal depression | −0.489 | † | 0.255 | 0.055 |  | −0.351 | * | 0.167 | 0.035 |
| ΔNoticing | 1^st^ Noticing | −0.506 | ** | 0.125 | 0.000 |  | −0.387 | ** | 0.147 | 0.009 |
| Intercepts |  |  |  |  |  |  |  |  |  |  |
|  | ΔPostnatal depression | 1.813 | ** | 0.672 | 0.007 |  | 2.592 | * | 1.114 | 0.020 |
|  | ΔNoticing | 1.361 | ** | 0.265 | 0.000 |  | 1.131 | ** | 0.324 | 0.000 |
|  | ΔPerceived stress | 20.677 | ** | 6.689 | 0.002 |  | 17.427 | * | 6.789 | 0.010 |
|  | Mothers’ valence | 12.924 |  | 15.399 | 0.401 |  | 20.365 | ** | 6.154 | 0.001 |
|  | Infants’ valence | 64.458 | ** | 13.084 | 0.000 |  | 65.223 | ** | 16.307 | 0.000 |
| Residual Variances |  |  |  |  |  |  |  |  |  |  |
|  | ΔPostnatal depression | 6.873 | ** | 2.429 | 0.005 |  | 10.199 | * | 3.936 | 0.010 |
|  | ΔNoticing | 0.427 | ** | 0.116 | 0.000 |  | 0.622 | ** | 0.180 | 0.001 |
|  | ΔPerceived stress | 28.116 | ** | 9.225 | 0.002 |  | 13.429 | ** | 3.422 | 0.000 |
|  | Mothers’ valence | 31.302 | ** | 11.393 | 0.006 |  | 8.531 | ** | 2.238 | 0.000 |
|  | Infants’ valence | 86.261 | ** | 19.756 | 0.000 |  | 86.580 | ** | 33.101 | 0.009 |

**The process of determining the participants.** The participants comprised healthy mothers and their first-born infants of 2–8 months who reported no diagnosis of mental illness or cardiovascular diseases. Oppenheimer et al. proposed that participants who are satisficing will often not bother to read the questions or instructions in a survey^1^. To deal with satisficing, the instructional manipulation check (IMC) is effective. IMC measures whether participants are reading the instructions, and thus provides an indirect measure of satisficing. In this survey, the IMC (“please choose option 3 for this question”) was added to the first and second session questionnaires. Participants who did not answer option 3 at least once to the IMC of the Sessions 1 and 2 questionnaires were excluded from the analysis. As the period between Sessions 1 and 2 was one month, assuming that the application was used once per day, it could be used about 30 times. In a previous study using the experience sampling method^2^, if more than 33% of the available data were not acquired, the acquired data were excluded from the analysis, so the exclusion criterion for this study was also set at 33% or more, and participants who used the application less than 11 times were excluded from the analysis. Among 173 participants in the first session, 20 did not participate in the second session, 21 mothers did not answer appropriately to the IMC, and 3 mothers in the IDS group used the application less than 11 times. Therefore, 129 mothers (mean age = 31.3 years, SD = 5.17) and infants (male: 67, mean age = 4.55 months, SD = 1.79) who participated in Sessions 1 and 2 were included in the analysis.

**Supplementary Table 6. The IDS Lyrics**

Lyrics, music, and song by “Tomoka Fujioka”

Japanese English

| おむつを替えよう ママと一緒に替えよう  キレイキレイ キレイキレイ 一緒にキレイキレイ オムツを替えよう ママと楽しく替えよう  キレイキレイ キレイキレイ 楽しくキレイキレイ  丸いおなかをナデナデ〜〜  パンパンおなかを プ〜〜〜 あ〜楽しいな！  おむつを替えよう ママと楽しく替えよう  キレイキレイ キレイキレイ 楽しくキレイキレイ  プリプリおしりをふきふき〜 サラサラおしりは「きもちいねぇ〜〜」  キレイキレイ  おむつを替えよう ママと一緒に替えよう  キレイキレイ キレイキレイ 一緒にキレイキレイ オムツを替えよう ママと楽しく替えよう  キレイキレイ キレイキレイ 楽しくキレイキレイ  柔らかほっぺをスリスリ〜 プニプニほっぺをハムハム〜〜 キレイキレイ | Let’s change your diapers; let’s change it together with mommy  Here, all nice and clean—together nice and clean  Let’s change your diapers; it’s so fun with mommy!  Here, so much fun it’s all nice and clean  Rubby rub your round belly,  Like a balloon, let me blow on it,  Oh so much fun!  Let’s change your diapers; it’s so fun with mommy!  Here, so much fun it’s all nice and clean  Wipey wipe your cute bottom  Smooth and dry feels so good!  Pretty and clean  Let’s change your diapers; let’s change together with mommy  Here, all nice and clean—together nice and clean  Let’s change your diapers; it’s so fun with mommy!  Here, so much fun it’s all pretty and clean  Squeezy squeeze those soft cheeks  Just makes me want to kiss you  So pretty and clean |
| --- | --- |

**Supplementary Table 7. Intra-class correlation coefficient (ICC) and confidence intervals for each variable of all samples in the no-IDS and IDS groups**

| Variable | N | ICC |  | 95% Lower | 95% Upper |
| --- | --- | --- | --- | --- | --- |
| Mothers’ HR | 5429 | .329 | ** | .253 | .432 |
| Mothers’ Calmness | 5390 | .367 | ** | .286 | .475 |
| Mothers’ Valence | 5390 | .392 | ** | .308 | .501 |
| Mothers’ Relaxation | 5390 | .319 | ** | .244 | .422 |
| Mothers’ Vitality | 5390 | .283 | ** | .213 | .381 |
| Infants’ Calmness | 5390 | .219 | ** | .161 | .306 |
| Infants’ Valence | 5390 | .287 | ** | .217 | .386 |
| Infants’ Relaxation | 5390 | .280 | ** | .211 | .378 |
| Infants’ Vitality | 5390 | .219 | ** | .161 | .305 |

***p* < .01

**

**

**Supplementary Figure 1. Multiple group multilevel structural equation model of the associations between the valence, maternal HR, and maternal interoceptive sensibility (noticing and trusting) of the mothers and infants. (a) No-IDS group model and (b) IDS group model.**

Solid line: positive effect; Broken line: negative effect. Estimation: maximum likelihood estimation, the path of notation unstandardized coefficients.

Note. Δ indicates the change score (Session 2 minus Session 1).

***p* < .01, **p* < .05, ^†^*p* < .10

An *a posteriori* explanatory analysis on the relationship between interoceptive sensibility and maternal valence was performed. A previous study has reported that facets of interoceptive sensibility and trait anxiety are associated mainly through the appraisal of one’s own body as safe and trustworthy^3^. We examined the possibility that the negative relationship between the awareness of body sensation (noticing) and the valence (negative affective states) of mothers in the no-IDS group could be mediated by a cognitive assessment (trusting) that the body of an individual is safe and trustworthy.

We conducted multiple group multilevel SEM with the data collected from the no-IDS and IDS groups to explore the causal structures of the associations between the valence, maternal HR, and maternal interoceptive sensibility (noticing and trusting) of the mothers and infants. In the between-levels analysis, the valence and maternal HR of the mothers and infants, Session 1 scores, and changes in each interoceptive sensibility scale were included. Additionally, changes in “noticing” and “trusting” were controlled by partialling out Session 1 scores for each scale. The valence and maternal HR of the mothers and infants were also included in the within-levels analysis. The robust maximum likelihood method was used for estimation. Furthermore, the ideal fit of the structural model was evaluated using standard indices, such as RMSEA and CFI, and Mplus version 8.466 was employed to perform the multiple group SEM.

Figure S1 shows the results of the multiple group multilevel structural equation modeling (SEM) analysis in the no-IDS and IDS groups. According to this model, the result of the χ2 test was not significant (χ2 (20) = 23.913, *p* = .246), with comparative fit index (CFI) = 0.991 and root mean square error of approximation (RMSEA) = 0.008, which is considered a good fit. Thus, it seemed that the model fits the data. In the no-IDS group, the increase in “noticing” affected the increase in “trusting,” and the increase in “trusting” affected the valence of the mothers. A significant difference was found between the no-IDS and IDS groups in the path coefficient of changes in “trusting” to the valence of the mothers (*p* = .019). Conversely, in the IDS group, the association between “noticing,” “trusting,” and the valence of the mothers was not significant.

This additional analysis suggested that in the no-IDS group, the awareness of body sensation may be mediated by the belief that the body is safe and an act in a direction that lowers pleasant feelings. This result may be explained by predictive coding theory^4^. “A high belief that the body is safe (trusting)” is considered to be a prediction from a higher-order inner model. The high “awareness of body sensation (noticing)” increases the stressful input associated with parenting, which causes a large prediction error between “trusting” and “noticing.” If the prediction error cannot be minimized, then the condition is thought to lead to the subjective experience of unpleasant feelings^5^.

**(a)**

| Participants report their own number of heartbeats. (25s) | rest | Participants report their own number of heartbeats. (35s) | rest | Participants report their own number of heartbeats. (45s) |
| --- | --- | --- | --- | --- |

| Participants wear an ECG to measure their own heart rate. |
| --- |

**(b)**

| The app counts the participants’ heart rate. (30s) | Participants enter their own number of heartbeats on the app. (30s) | rest | The app counts the participants’ heart rate. (30s) | Participants enter their own number of heartbeats on the app. (30s) | rest | The app counts the participants’ heart rate. (30s) | Participants enter their own number of heartbeats on the app. (30s) |
| --- | --- | --- | --- | --- | --- | --- | --- |

**Supplementary Figure2. Experimental procedures of (a) HCT-C and (b) HCT-A.**

**Supplementary Figure 3. The relationship between the heartbeat perception score of HCT-C and HCT-A.**

**The verification of the reliability and validity of HCT-A**

Twenty participants (male: 10, age = 20.0 ± 1.47 years) performed the heartbeat-counting task^6^, which measured the accuracy of interoceptive sensitivity, and a smartphone version of the heartbeat-counting task on the application (HCT-A). The experimental protocol is shown in Supplementary Figure 2.

Initially, electrodes to measure the electrocardiogram (ECG) were attached to the participants. Next, participants performed the conventional heartbeat-counting task (HCT-C) based on the methods of Schandry (1981)^6^. They were asked to sit on a chair in the laboratory chamber and instructed not to take a cue by taking a pulse with their bodies. They counted the number of heartbeats in each of the three sections (25s, 35s, and 45s) and reported it after each section. Then, they performed the heartbeat-counting task on the smartphone application (HCT-A). Using the same instruction as HCT-C, they held their smartphone in both hands and counted the number of heartbeats in 30 s and entered it on the application. After that, the heart rate was measured on the application, and the heart rate per minute was calculated. As with the HCT-C, they performed this procedure three times.

To verify the reliability and validity of the HCT-A, we calculated the heartbeat perception scores of the HCT-C and HCT-A based on Schandry (1981)^6^. The heartbeat perception score is calculated according to the following equation:

$$Hearbeat perception score= \frac{1}{3}\sum_{i=1}^{3} \left( 1-\frac{\left| na_{i}-nr_{i} \right|}{na_{i}} \right)$$

$i$ is the number of sections,$na$is the number of actual heartbeats, and $nr$is the number of reported heartbeats^7^.

The heartbeat perception score is a value ranging from 0 to1, the closer to 1 the score, the more accurately the participant was able to perceive his or her own heartbeat. To calculate the heartbeat perception score of the HCT-C, heart rate measured by ECG and the participant’s own heart rate was used. To calculate the heartbeat perception score of the HCT-A, the heart rate measured on the application and the number of heartbeats entered by the participants on the application were used. The heartbeat perception score of the HCT-C was 0.738 ± 0.169. The heartbeat perception score of the HCT-A was 0.715 ± 0.133.

The correlation analysis indicated that there was a positive correlation between the HCT-C and the HCT-A heartbeat perception scores (*r* (18) =.667, *p* =.001) (Supplementary Figure 3). Therefore, the HCT-A had sufficient validity. To verify the reliability of HCT-S, the Cronbach’s α for the HCT-A score across sections was calculated. The results indicate that the HCT-A had sufficient reliability (*α* =.842).

**References**

1 Oppenheimer, D. M., Meyvis, T. & Davidenko, N. Instructional manipulation checks: Detecting satisficing to increase statistical power. *J. Exp. Soc. Psychol.* **45**, 867-872 (2009).

2 Kimhy, D. *et al.* Computerized experience sampling method (ESMc): assessing feasibility and validity among individuals with schizophrenia. *J. Psychiatr. Res.* **40**, 221-230 (2006).

3 Slotta, T., Witthöft, M., Gerlach, A. L. & Pohl, A. The interplay of interoceptive accuracy, facets of interoceptive sensibility, and trait anxiety: A network analysis. *Pers. Individ. Dif.* **183** (2021).

4 Barrett, L. F. The theory of constructed emotion: an active inference account of interoception and categorization. *Soc. Cogn. Affect. Neurosci.* **12**, 1-23 (2017).

5 Barrett, L. F. & Simmons, W. K. Interoceptive predictions in the brain. *Nat. Rev. Neurosci.* **16**, 419-429 (2015).

6 Schandry, R. Heart beat perception and emotional experience. *Psychophysiology* **18**, 483-488 (1981).

7 Werner, N. S. *et al.* Interoceptive awareness moderates neural activity during decision-making. *Biol. Psychol.* **94**, 498-506 (2013).
