# Supplementary figures and images for: Mothers’ interoceptive sensibility mediates affective interaction between mother and infant
Source: Sci Rep. 2022 Apr 15;12:6273. doi: 10.1038/s41598-022-09988-y (PMC9011379; doi:10.1038/s41598-022-09988-y)

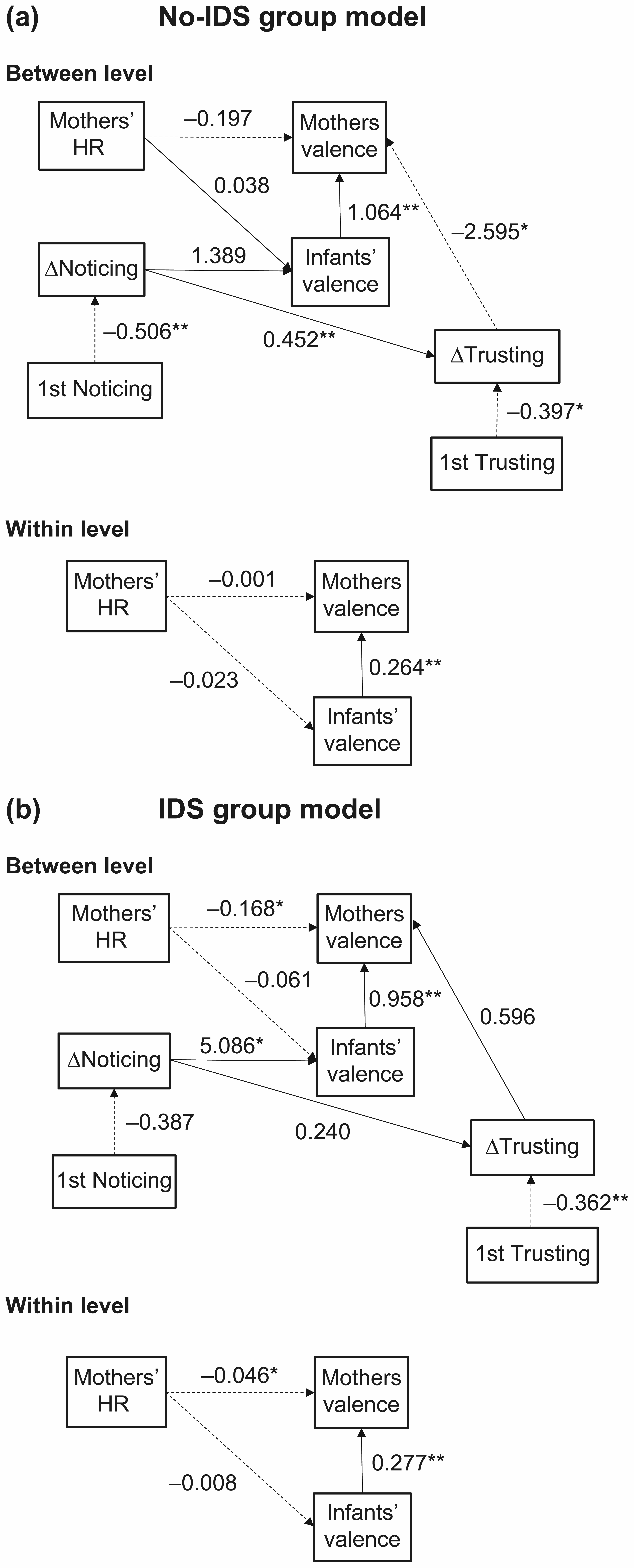

Supplement: Supplementary file 2 — Supplementary Figure 1. [file 41598_2022_9988_MOESM2_ESM.tif]

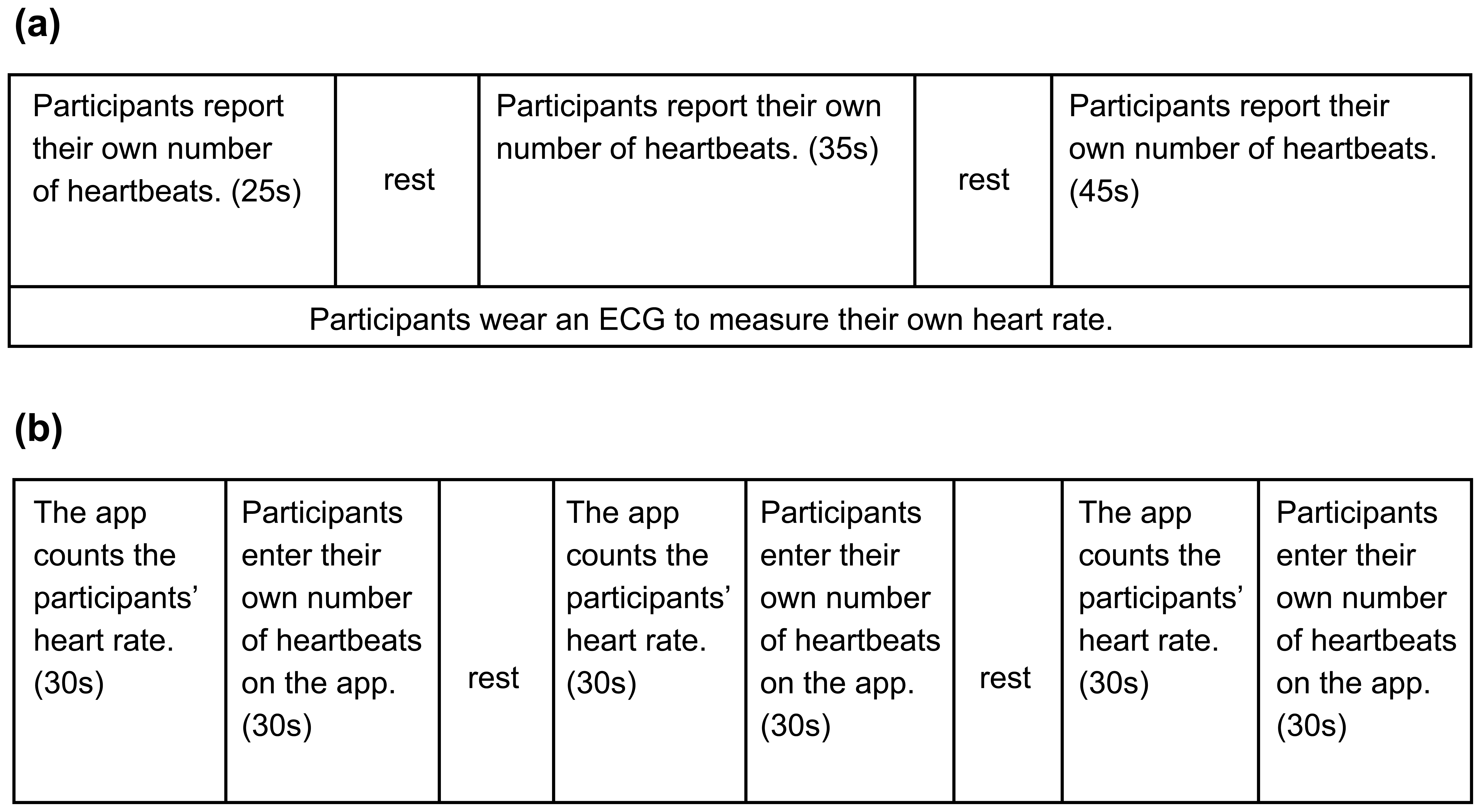

Supplement: Supplementary file 3 — Supplementary Figure 2. [file 41598_2022_9988_MOESM3_ESM.tif]

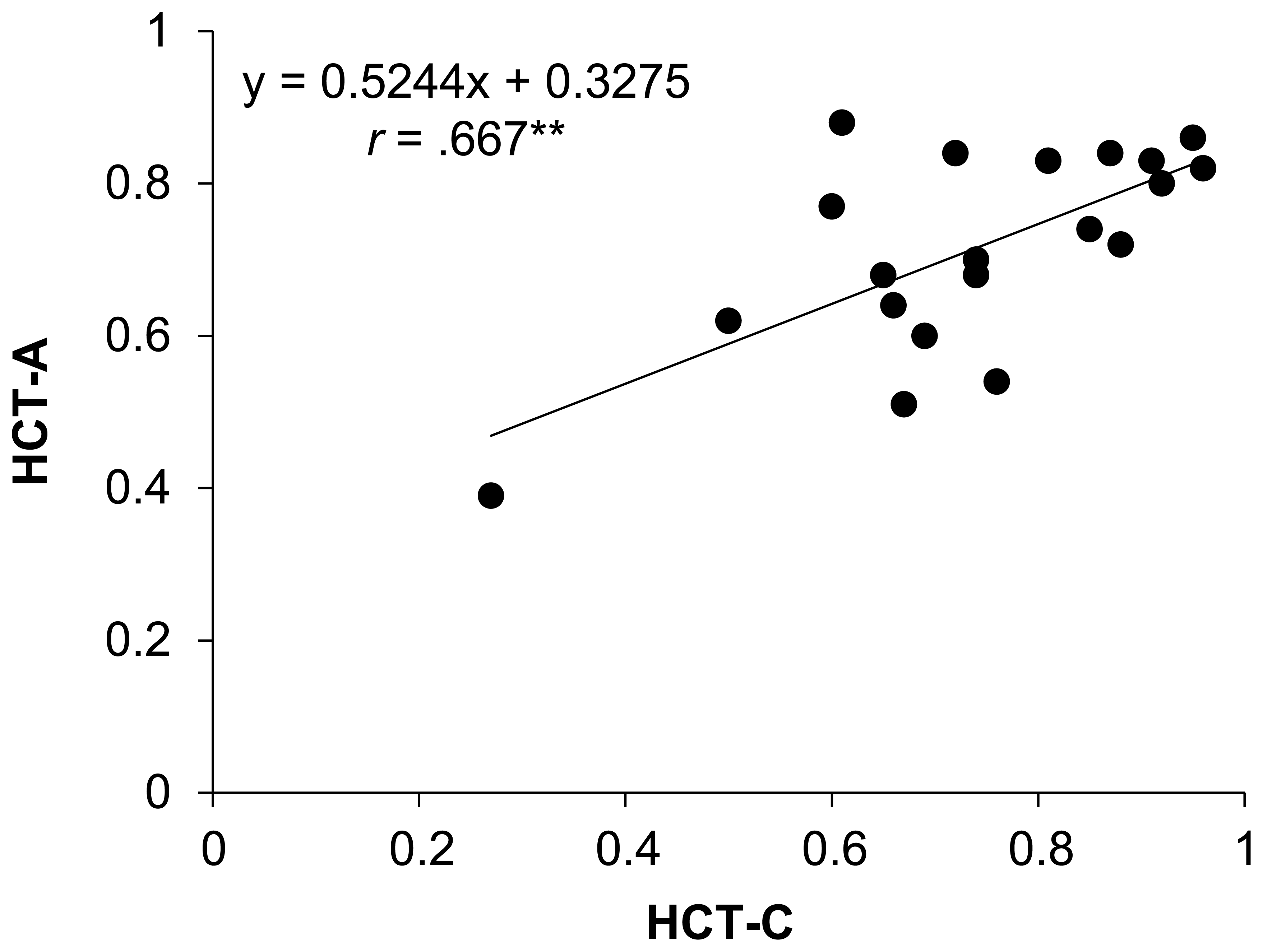

Supplement: Supplementary file 4 — Supplementary Figure 3. [file 41598_2022_9988_MOESM4_ESM.tif]
